# Retinal and choroidal blood flow changes in dialysis patients assessed by wide-field swept-source optical coherence tomography angiography

**DOI:** 10.3389/fmed.2025.1524503

**Published:** 2025-05-14

**Authors:** Wan Li, Qing Zhou, Jiulin Ni, Xun Pan, Min Li, Fei Hua, Huan Tang, Shuting Li

**Affiliations:** ^1^Department of Ophthalmology, The First People’s Hospital of Changzhou, Changzhou, China; ^2^Department of Ophthalmology, Changzhou Municipal Hospital of Traditional Chinese Medicine, Changzhou, China; ^3^Department of Nephrology, The First People’s Hospital of Changzhou, Changzhou, China; ^4^Department of Endocrinology, Changzhou No.1 Hospital, The Third Affiliated Hospital of Soothe First People’s Hospital of Changzhou, Changzhou, China

**Keywords:** hemodialysis, swept-source optical coherence tomography angiography (SS-OCTA), blood flow, choroid, perfusion area

## Abstract

**Purpose:**

The study utilized wide-field swept-source optical coherence tomography angiography (SS-OCTA) to examine the alterations in retinal and choroidal blood flow in patients with end-stage renal disease (ESRD) before and after hemodialysis.

**Methods:**

Forty-two eyes of 24 participants with end-stage renal disease were enrolled in this prospective and cross-sectional study. A full ophthalmic examination was conducted on each participant 1 h before and after hemodialysis, which included wide-field SS-OCTA imaging on OCTA scans measuring 9 mm x 9 mm. The mean perfusion area (PA), superficial vessel density (SVD), retinal inner VD (RIVD), and choroidal vascularity index (CVI) were evaluated independently before and after hemodialysis in both macular and optic disk areas across all 16 sectors defined by the Early Treatment Diabetic Retinopathy Study (ETDRS).

**Results:**

The study subjects consisted of 18 men and 6 women, comprising 9 patients with diabetic renal failure and 23 patients with hypertension. The mean age of the patients with end-stage renal disease was 57.3 ± 12.7 years. In the central ring, retinal inner VD were significantly decreased after HD (*p* < 0.05), but there were no significant differences in the average PA,SVD, and CVI between before and after single HD session. In the 3 mm radius, inferior quadrant of macular retinal inner VD, PA of the macular and optic disk were significantly decreased after HD (*p* < 0.05). In the 6 mm radius, the temporal section of the retinal inner VD, nasal and inferior of PA were noticeably reduced in the HD group. In the wide-field scans (9 mm radius), there was exhibited a more pronounced reduction in inferior PA, SVD and retinal inner VD of optic disk area in inferior sectors after HD. Fairly consistent CVI values were noted in the macular zone, except for a statistically significant decrease in the temporal quadrant at 3 mm and 6 mm within the optic disk area (*p* = 0.040 and 0.048, respectively).

**Conclusion:**

Results revealed a more significant decrease in indicators of the choroid and optic disk area compared to the inner retina and macular zone. Wide-field SS-OCTA displays potential as an imaging technique for early detection of microcirculation changes in hemodialysis patients.

## Introduction

End-stage renal disease (ESRD) seriously impacts the quality of life of patients (1). The incidence of ESRD is progressively rising, with projections expecting the number of affected individuals in China to reach 4.8 million by 2032 (2). ESRD can cause the functional decline of many important organs and systems, resulting in various complications. Studies have identified ocular issues in ESRD patients, such as macular edema, ischemic optic neuropathy, elevated intraocular pressure, retinal detachment, and retinal hemorrhage (3, 4). Hemodialysis (HD) is a common outpatient, hospital, nursing facility, and home-based treatment known for its safety in clinical practice (5–8). The mechanism of HD is to remove excess water and toxins from the body, maintaining blood volume balance (9). Ocular alterations associated with HD encompass refractive changes, intraocular pressure fluctuations, changes in the corneal endothelium, central corneal thickness, retinal nerve fiber layer thickness, and choroidal thickness. Reports on these changes before and after HD yield controversial results. Elbay et al. (10) conducted that subfoveal choroidal thickness decreased after HD. Shoshtari et al. (11) and Kal et al. (12) also found decreasing choroidal thickness, but the retinal thickness did not change after HD. However, Atilgan et al. (13) thought that macular and retinal nerve fiber layer (RNFL) thicknesses of patients receiving HD were less than the pre-dialysis population, which was contradictory with Shoshtari et al. (11). Zegrari et al. (14) concluded that non-perfusion in the choriocapillaris increased significantly using optical coherence tomography angiography (OCTA), while no significant modification of the vascular density was observed in the retinal vasculature around the macular zone or the optic nerve. Metabolite concentration variations during HD, like blood urea, sodium, potassium, albumin, and glucose levels, may induce changes in ocular parameters. These changes can induce alterations in ocular parameters (11, 12). However, the impact of HD on retinal and choroidal blood flow alterations remains uncertain.

Optical coherence tomography angiography (OCTA) is a non-invasive imaging technique that assesses blood flow by detecting the movement of erythrocytes within vessels, along with providing three-dimensional imaging of the choroidal and retinal microvascular layers (15). Ultra-wide-angle swept-source optical coherence tomography angiography (SS-OCTA) offers various advantages such as increased scanning speed, enhanced signal intensity, and deeper tissue penetration (16). Its high-resolution allows for the straightforward visualization of retinal and choroidal blood flow without the need for contrast agents, particularly crucial for patients with renal failure. Importantly, capillary changes can be observed even in the early stages (17).

In this study, SS-OCTA was utilized to observe and quantitatively analyze retinal and choroidal microvascular parameters, including perfusion area (PA), vessel density (VD), and choroidal vascularity index (CVI). These parameters were compared across different macular and disk regions in ESRD patients before and after HD. The aim of this study was to use SS-OCTA to investigate the alterations of retinal and choroidal blood flow in patients with renal failure before and after HD.

## Patients and methods

This observational study conducted to investigate the effects of hemodialysis (HD) on ocular parameters in patients with kidney failure. It was conducted in accordance with the Declaration of Helsinki guidelines and approved by the Medical Research and Ethics Committee of The Third Affiliated Hospital of Soochow University. Informed consent was obtained from all participants before their inclusion in the study.

The study included 24 patients undergoing HD for various causes of kidney failure, such as glomerular disease, nephrotic syndrome, diabetic nephropathy, and polycystic kidney disease between September 2023 and March 2024.Both before and after HD, participants underwent a comprehensive ophthalmologic examination. This included a detailed medical history, assessment of visual acuity (VA), refraction, intraocular pressure (IOP), anterior segment biomicroscopy, wide-field color fundus imaging, and wide-field swept-source optical coherence tomography angiography (SS-OCTA) (VG200S; SVision Imaging, Ltd., Henan, China). Demographic information, including age, gender, weight, diabetes mellitus, and hypertension status, was collected for all participants. Additionally, serum creatinine (Cr) levels and estimated glomerular filtration rates (eGFR) were recorded.

Inclusion Criteria: Participants were required to be over 18 years of age and diagnosed with end-stage renal disease (ESRD), undergoing hemodialysis (HD) therapy three times weekly. Exclusion Criteria: Individuals under 18 years of age, individuals with a refractive error of −6.00 diopters or greater, or +3 diopter-equivalent spheres, serious systemic diseases (such as tumors, strokes, or dementia), macular edema, a history of ocular trauma or vitreoretinal surgery, certain ocular conditions, an inability to provide informed consent or undergo a comprehensive examination, and low-quality imaging (quality index < 6 or images with excessive motion artifacts) were excluded from the study.

The SS-OCTA images were obtained using a scanning range of 9 × 9 mm with 768 A-scan per B-scan (24 mm spacing between adjacent A-scans) and 768 B-scan positions per volume scan (24 mm spacing between adjacent B-scans). OCTA images were produced by acquiring four replicated B-scans at each B-scan spot. The scan depth was 3 mm. The equipment utilized eye-tracking technology to minimize motion artifacts. The scans’ quality scores were expressed as an SNR in decibels (dB) on a scale of 1 (poor quality) to 10 (excellent quality), with scans scoring>6 dB considered good quality and included. All examinations were performed by an experienced professional ophthalmic technician.

**Figure 1:**
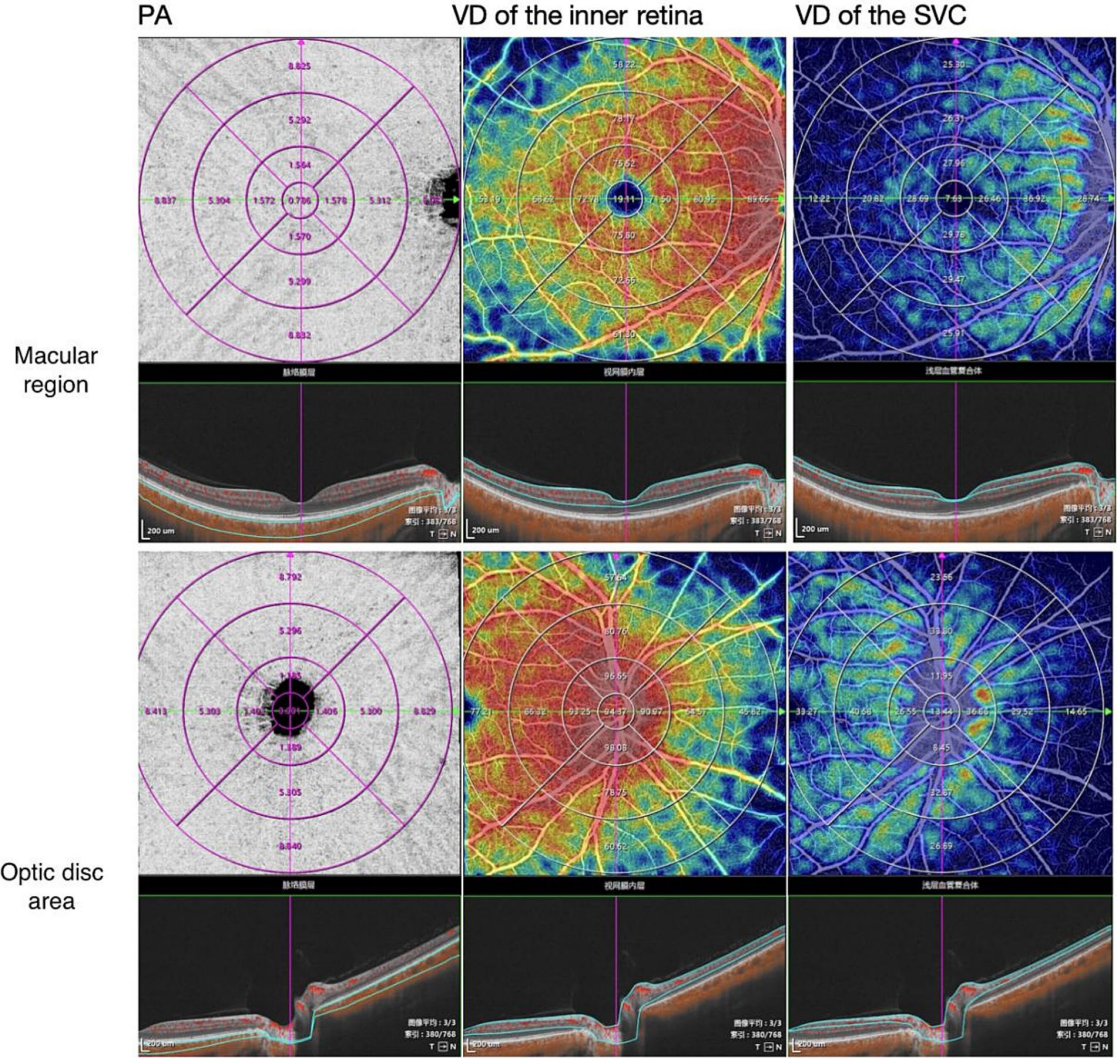
Representative en face views of SS-OCT/OCTA scans are shown with the following components: PA (Perfusion Area), VD (Vessel Density) of the inner retina, and VD of the Superficial Vessel Capillary (SVD) using the ETDRS grid. SS-OCT/OCTA stands for swept-source optical coherence tomographic angiography. The scans provide insights into the perfusion area and vessel density of the inner retina and superficial vessel capillary.

**Figure 2:**
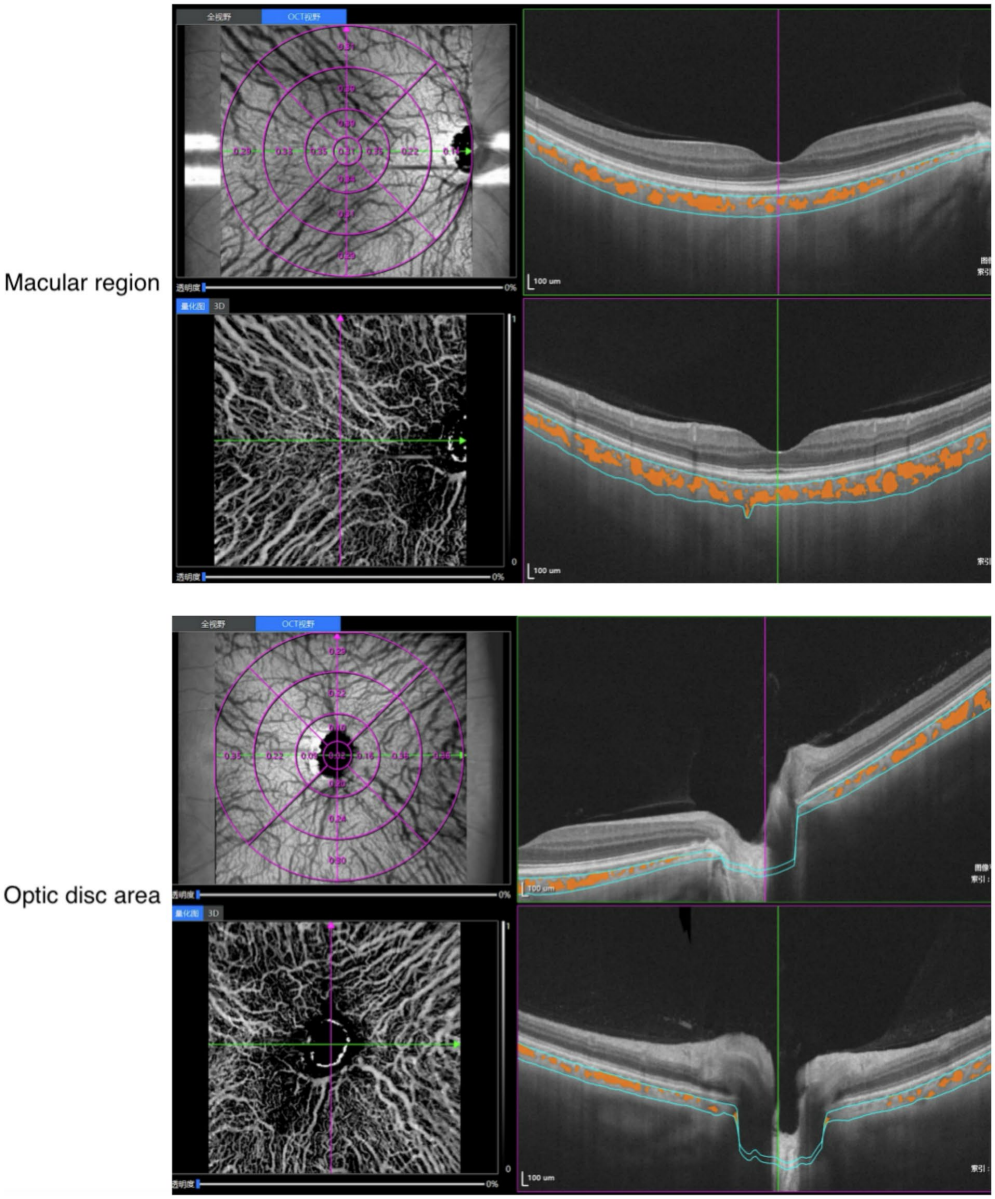
Representative SS-OCT/OCTA scans in en face view with CVI (ETDRS grid); CVI, choroidal vascularity index; SS-OCT/OCTA, swept-source optical coherence tomographic angiography.

Choroidal and retinal parameters, such as perfusion area (PA), vessel density (VD), and choroidal vascular index (CVI), were calculated in different annular regions before and after HD separately. The processed images are shown in Figures 1, 2 with built-in software of VG200, SVision Imaging (version 3.0.184.). Topographic measurements were performed using the built-in software of the VG200 device, including Early Treatment Diabetic Retinopathy Study (ETDRS) subfield analysis. And, the data were automatically averaged through the following subfields and sectors: the central fovea subfield within the inner 1-mm-diameter circle; the circle subfield between (1–3 mm)-diameter circles, (3–6 mm)-diameter circles and (6–9 mm)-diameter circles. Each circle was sectioned into nasal, temporal, superior and inferior quadrants.

Analysis of retinal subfields and sectors, as well as the inner retina (from ILM-5 to INL/2) and superficial vessel complex (SVC) (from ILM-5 to NFL/GCL), was performed using the built-in software of the VG200 device (version 3.0.184; VG200D, SVision Imaging, Ltd). Besides, we measured and automatically calculated the total choroidal area (TCA) and luminal area (LA) of the subjects under built-in system. Choroidal vascular index (CVI) is defined as the ratio or proportion of the LA within the TCA (see Figure 2).

### Statistical analysis

Using SPSS software (version 26.0 SPSS, IBM Corporation, Chicago, IL, United States) to conduct all statistical analyses in this study. Shapiro–Wilk tests were conducted to validate data normality before use of paired *t*-test. All the variables were expressed as the mean ± standard deviation (SD). The paired t test was used to compare the variables before and after hemodialysis. An analysis was performed to determine if hemodialysis-induced changes in perfusion area (PA), superficial vessel density (SVD), retinal inner VD (RIVD), and choroidal vascularity index (CVI) were correlated with baseline age, blood glucose, serum creatinine, body weight, Baseline SBP, DBP, the change in SBP, DBP and body weight. Correlations between the measurements were analyzed by Pearson’s correlation test.

## Results

The demographic characteristics of the study participants are presented in Table 1. A total of 24 individuals, contributing 42 eyes, were included in this study. Two eyes with high myopia (had a refractive error within −6.00 equivalent spheres), one eye had history of branch retinal vein occlusion and three eyes had diabetic retinopathy were excluded. The male-to-female ratio was 18: 6, with a mean patient age of 57.3 ± 12.7 years. Various etiologies were identified as leading to kidney failure, with nine participants having diabetes mellitus and 23 having hypertension. Subjects experienced a decrease in weight from 64.48 ± 2.53 to 62.20 ± 2.41 before and after hemodialysis (HD). Although both systolic and diastolic blood pressure showed slight increments post-HD, the differences were not statistically significant. Utilizing an averaging mode to mitigate noise sensitivity, the quantification analyses revealed no significant differences in parameters related to signal and image quality.

**Table 1:** Demographic data of patients.

General baseline data	
Number of patients (female)	24 (6)
Eyes (right)	42 (22)
Age (years)	57.3 ± 12.7
Duration of HD (months)	
The causes of kidney failure	
Diabetic nephropathy	9
Focal segmental glomerular sclerosis (FSGS)	2
Membranous nephropathy (MN)	1
Polycystic kidney	2
Hydronephrosis	2
Other causes	8
The proportion of DM (%)	37.5
The proportion of hypertension (%)	95.83
Weight (kg)	
Before HD	64.48 ± 2.53
After HD	62.20 ± 2.41
SBP (mmHg)	
Before HD	146.04 ± 16.35
After HD	146.33 ± 20.04
DBP (mmHg)	
Before HD	80.92 ± 13.27
After HD	83.92 ± 14.71

Values are presented as the means ± standard deviations at baseline. DM, diabetes mellitus; HD, hemodialysis; SBP, systolic pressure; DBP, diastolic pressure.

### Comparison of the PA in the macular zone and optic disk area

**Figure 3:**
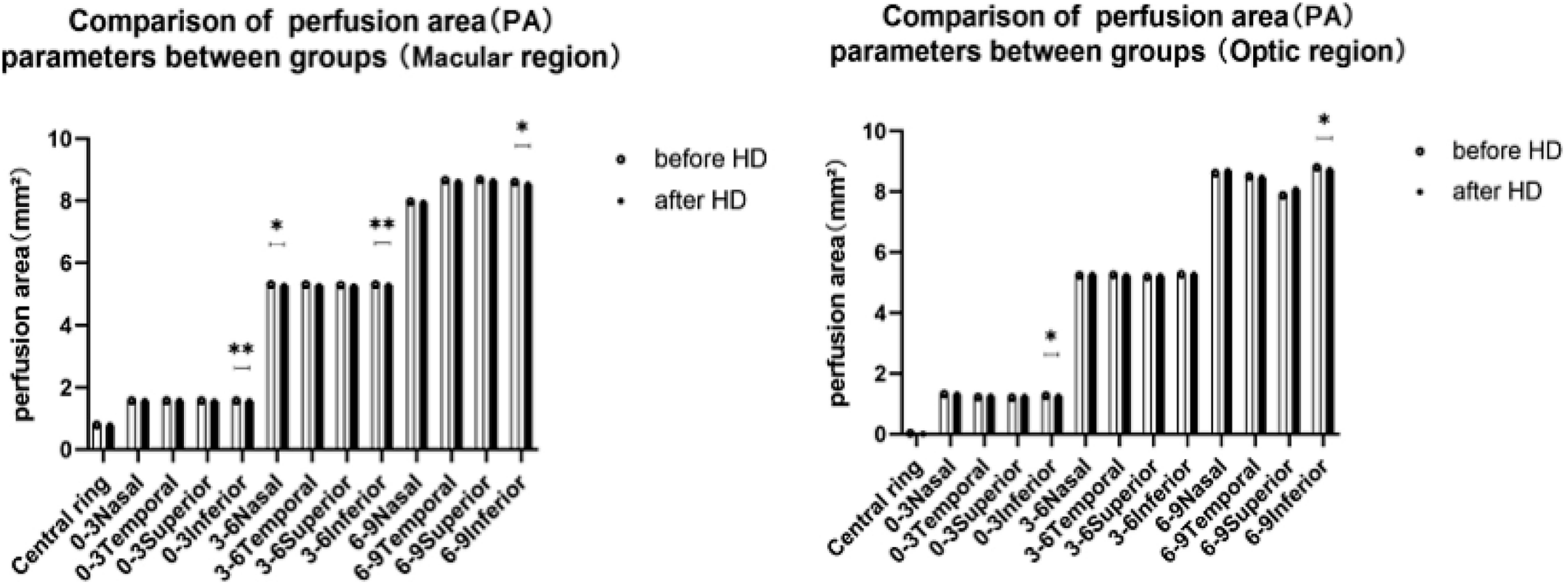
Comparison of PA between groups in the macular region and optic region. * indicates a statistically significant difference (*p <* 0.05). ** indicates a statistically significant difference (*p* < 0.01). PA, perfusion area; HD, hemodialysis.

**Figure 4:**
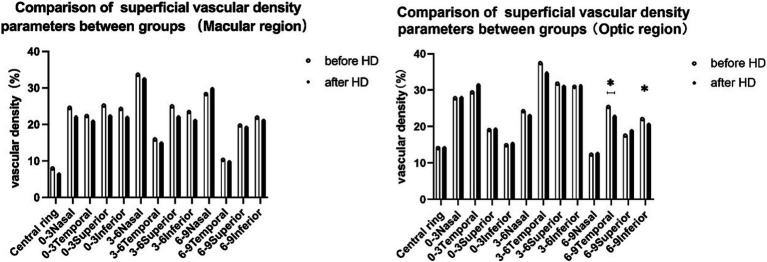
Comparison of SVD between groups in both the macular and optic regions. * indicates a statistically significant difference (*p* < 0.05). SVD, vessel density of the superficial vessel capillary; HD, hemodialysis.

A comparison of perfusion area (PA) between the macular zone and optic disk area revealed distinct findings. The PA values for patients before and after hemodialysis (HD) in these regions were depicted in Figure 3. Statistical analysis of variance indicated a notable decline in the mean PA of the macular region across all quadrants and ranges following HD. Specifically, the inferior quadrant of 3 mm (from 1.571 ± 0.003 to 1.551 ± 0.096) and 6 mm (from 5.300 ± 0.004 to 5.282 ± 0.061) exhibited significantly reduced perfusion areas post-hemodialysis (*p* < 0.01, refer to Figure 3). Additionally, the nasal quadrant of 6 mm (from 5.301 ± 0.009 to 5.271 ± 0.096) and the inferior quadrant of 9 mm (from 8.605 ± 0.393 to 8.527 ± 0.424) also displayed significantly diminished perfusion areas (*p* < 0.05, see Figure 3). Substantial decreases in PA were also observed in the optic disk region, with the PA values of the 3 mm and 9 mm inferior quadrants both showing significant decreases post-HD compared to pre-HD values (*p* = 0.039 and 0.048, respectively). With the exception of the 6–9 mm scale, which saw a significant increase in the nasal quadrant after HD (*p* = 0.018, Figure 4), no other quadrants in the macular region exhibited growth. Figure 4 elaborates on the SVD parameters of the optic disk area across different ranges.

### Comparison of the SVD in the macular zone and optic disk area

With the exception of the 6–9 mm scale, in which the nasal quadrant showed a significant increase after hemodialysis (HD) (*p* = 0.018; see Figure 4), there were no notable changes observed in the other quadrants of the macular region. Figure 4 also presents the SVD parameters for the optic disk area across different ranges. When comparing the SVD values before and after HD for each region, statistically significant changes were only evident in the 6–9 mm range within the optic disk area. Specifically, the SVD values in the temporal and inferior quadrants of the 6–9 mm region exhibited significant decreases following HD (from 25.38 ± 9.91 to 22.84 ± 10.08 and 22.05 ± 5.43 to 20.61 ± 5.67, respectively; *p* = 0.035 and 0.047).

### Comparison of the retinal inner VD in the macular zone and optic disk area

The retinal inner vessel density (VD) in the macular region showed a decreasing trend in all regions (refer to Table 2). Furthermore, statistically significant decreases were observed in the inferior quadrant of 3 mm, as well as in the temporal quadrants of 6 mm and 9 mm (*p* = 0.039, 0.013, and 0.036, respectively). In the central ring, the retinal inner VD of patients after HD was significantly lower than that before HD in the optic disk area (*p* = 0.014). Additionally, notable differences were found in the temporal quadrant of 6 mm, the inferior quadrant of 6 mm, and the inferior quadrant of 9 mm (*p* = 0.019, 0.040, and 0.022, respectively), as shown in Table 2.

**Table 2:** Comparison of retinal inner vessel density (VD) parameters before and after HD.

Retinal inner VD	Macular region			Optic disk area		
	Before HD	After HD	*P*	Before HD	After HD	*P*
Central ring	15.60 ± 8.54	13.12 ± 6.14	0.057	88.69 ± 8.73	85.60 ± 11.36	0.014*
3 mm radius						
Nasal	55.65 ± 21.24	51.13 ± 19.93	0.158	82.20 ± 10.81	82.80 ± 9.57	0.251
Temporal	53.77 ± 20.08	52.35 ± 18.22	0.293	89.30 ± 8.05	87.66 ± 9.08	0.099
Superior	58.22 ± 20.39	53.96 ± 22.47	0.053	92.43 ± 6.62	92.12 ± 7.05	0.315
Inferior	58.52 ± 20.47	53.40 ± 21.81	0.039*	93.55 ± 6.43	93.62 ± 7.56	0.670
6 mm radius						
Nasal	71.16 ± 17.83	68.79 ± 17.48	0.191	50.89 ± 14.87	50.35 ± 15.30	0.863
Temporal	52.29 ± 15.89	50.57 ± 16.84	0.013*	73.61 ± 15.60	67.70 ± 17.36	0.019*
Superior	61.34 ± 14.07	57.83 ± 16.29	0.099	68.00 ± 11.22	67.71 ± 12.61	0.826
Inferior	58.46 ± 15.10	54.36 ± 17.84	0.069	72.01 ± 11.27	70.06 ± 12.33	0.04*
9 mm radius						
Nasal	83.25 ± 9.97	81.97 ± 11.82	0.813	33.04 ± 13.57	34.81 ± 12.68	0.322
Temporal	38.24 ± 12.68	35.87 ± 13.21	0.036*	57.50 ± 18.63	53.01 ± 18.41	0.067
Superior	45.75 ± 13.14	45.17 ± 15.04	0.694	38.14 ± 8.72	40.55 ± 11.98	0.483
Inferior	49.99 ± 11.10	48.76 ± 10.77	0.531	48.44 ± 10.48	45.32 ± 12.18	0.022*

Retinal inner VD, retinal inner vessel density; *p* < 0.05 indicates a statistically significant difference, annotated with *.

### Comparison of the CVI in the macular zone and optic disk area

The CVI of the macular region exhibited a general decrease across various regions, although none of the changes in individual quadrants were statistically significant (refer to Table 3). The CVI values for the optic disk area in each region are also presented in Table 3. Although there was considerable variability in the CVI values of patients after HD compared to those before HD, significant lower CVI values were observed in the temporal quadrant of 3 mm and 6 mm (*p* = 0.040 and 0.048, respectively).

**Table 3:** Comparison of CVI parameters before and after HD (macular region and optic disk area).

CVI	Macular region			Optic disk area		
	Before HD	After HD	*P*	Before HD	After HD	*P*
Central ring	0.389 ± 0.072	0.382 ± 0.082	0.346	0.005 ± 0.014	0.006 ± 0.012	0.211
3 mm radius						
Nasal	0.375 ± 0.083	0.362 ± 0.094	0.284	0.187 ± 0.074	0.179 ± 0.075	0.203
Temporal	0.372 ± 0.060	0.367 ± 0.067	0.455	0.172 ± 0.073	0.159 ± 0.074	0.040*
Superior	0.379 ± 0.069	0.366 ± 0.083	0.373	0.167 ± 0.058	0.160 ± 0.064	0.168
Inferior	0.378 ± 0.090	0.368 ± 0.095	0.614	0.146 ± 0.066	0.133 ± 0.065	0.214
6 mm radius						
Nasal	0.326 ± 0.095	0.320 ± 0.102	0.434	0.333 ± 0.122	0.335 ± 0.121	0.767
Temporal	0.319 ± 0.066	0.315 ± 0.066	0.985	0.332 ± 0.110	0.324 ± 0.120	0.048*
Superior	0.338 ± 0.070	0.332 ± 0.070	0.652	0.299 ± 0.092	0.288 ± 0.107	0.223
Inferior	0.343 ± 0.091	0.350 ± 0.092	0.864	0.257 ± 0.104	0.253 ± 0.112	0.480
9 mm radius						
Nasal	0.225 ± 0.086	0.219 ± 0.087	0.281	0.351 ± 0.084	0.358 ± 0.081	0.463
Temporal	0.293 ± 0.052	0.292 ± 0.051	0.850	0.388 ± 0.075	0.368 ± 0.089	0.062
Superior	0.311 ± 0.069	0.308 ± 0.060	0.767	0.286 ± 0.075	0.301 ± 0.079	0.306
Inferior	0.324 ± 0.084	0.313 ± 0.093	0.773	0.277 ± 0.106	0.268 ± 0.112	0.163

CVI, choroidal vascularity index, *p* < 0.05 indicates a statistically significant difference, annotated with *.

### Comparison of the retinal thickness and choroidal thickness before and after HD

There was no significant difference between the cent retinal thicknesses before and after HD (*p* > 0.05 for all). Except for the choroidal thickness in subfoveal, temporal and nasal of 3 mm quadrants, nasal of 6 mm quadrants, the choroidal thickness in most regions of the patients significantly decreased after dialysis. There were significant differences between the choroidal thicknesses before and after HD in the 9 mm quadrants of optic disk area (*p* < 0.05 for all). Sectored analyses of CT in the ETDRS grid are shown in Supplementary Tables 3, 4.

## Discussion

This study investigates the changes in retinal and choroidal blood flow in patients with ESRD before and after HD using SS-OCTA. It was widely acknowledged that OCTA enables a non-invasive evaluation of microcirculation within the retina, optic disk, and partially in the choroid. Therefore, OCTA was often used clinically in systemic diseases to monitor changes in fundus circumstance. Previous studies (18) have indicated that patients with conditions such as hypertension, diabetes mellitus, kidney disease, preeclampsia, coronary artery disease, carotid artery stenosis, chronic obstructive pulmonary disease (19), and obstructive sleep apnea syndrome often exhibit lower retinal and choroidal vessel density (VD) and length (VL), alongside an increased foveal avascular zone (FAZ) area and perimeter. Alé-Chilet et al. (20) demonstrated that OCTA parameters like vessel density (VD) and foveal avascular zone circularity (FAZc) could discern different categories of diabetic kidney disease (DKD) severity and the risk of DKD progression in Type 1 Diabetes Mellitus (T1DM) patients. That the OCTA parameters. Yong et al. (21) also underscored the utility of OCTA in patients with Chronic Kidney Disease (CKD), revealing significant reductions in VD and perfusion density (PD) among individuals with CKD.

The investigation into changes in eye parameters before and after HD presents an intriguing topic for further exploration. Sariyeva Ismayılov et al. (22) highlights significant changes in eye parameters before and after HD, indicating a decline in intraocular pressure (IOP) and anterior chamber depth (ACD), along with a significant increase in axial length (AL) post-HD. Sun et al. (23) observed a significant dilation in retinal arteriolar caliber (RAC) and retinal venular caliber (RVC) following HD, signifying potential changes in retinal microvasculature. These findings suggest that patients with ESRD undergoing HD may experience alterations in systemic microvascular function, warranting further investigation into the ocular implications of HD in this patient population. Understanding how HD might impact factors such as IOP, ACD and retinal vascular caliber could provide valuable insights into the ocular effects of this medical intervention. Despite the increasing number of studies on this topic, to our knowledge, no previous study has specifically investigated alterations in PA, VD, and CVI before and after hemodialysis using the wide-field (9 mm × 9 mm) scans.

The OCTA assessments provide a non-invasive, efficient, and reproducible approach for evaluating both choroidal thickness (CT) and ocular microcirculation. Importantly, they eliminate the need for contrast agents, which is vital for patients with renal impairment. Several informative OCTA parameters can be utilized to evaluate diabetic retinopathy (DR) in clinical trials, such as measurements of the FAZ, VD, extrafoveal avascular zones and neovascularization. Considering emerging evidence highlighting the significance of the peripheral retinal vasculature in appraising and predicting DR advancement, wide-field OCTA imaging should be used (24).

Suciu et al. (25) compiled findings from studies investigating specific OCT (angiography) parameters related to diabetic macular edema, including central subfoveal thickness (CST), choroidal thickness (CT), choroidal vascularity index (CVI), vessel density (VD), and superficial capillary plexus (SCP). Their objective was to synthesize current biomarkers utilized for early diagnosis, assessment, monitoring, and prognosis prediction. They concluded that advancements in OCT and OCTA imaging techniques have novel parameters with potential as diabetes biomarkers. Furthermore, Yang et al. (26) noted that focusing on inner retinal blood flow density and central retinal blood flow density within the 3 × 3 OCTA window enables a more sensitive prediction of diabetic retinopathy onset in diabetic individuals. In comparison to studies on retinal conditions like DM and hypertension, the use of OCTA in hemodialysis patients with kidney disease remains underexplored.

The blood vessels of the retina and kidney has similar microvascular system, suggesting that ophthalmological assessments could provide valuable insights into the complex hemodynamic and neurohormonal changes associated with ESRD (27). In our study, we used the 9 × 9 mm ETDRS grid protocol instead of the smaller 3 × 3 mm or 4.5 × 4.5 mm grids used in prior research, enabling earlier detection of significant changes. Our assessment included measuring PA and CVI, a novel marker for choroidal assessment, to evaluate alterations in choroidal blood flow post-HD, expected to mirror systemic blood flow changes. Both of them decreased after HD in several quadrants. Consistent with our findings, Zegrari et al. (14) observed a significant increase in non-perfusion in the choriocapillaris post-HD (41.65 ± 3.58 before HD, 42.95 ± 3.19 after HD, *p* = 0.036). Lahme et al. (28) further supported these observations, noting a notable reduction in whole en-face flow density of the superficial capillary plexus (SCP) and choriocapillaris in the HD group compared to the control group. Evaluating ocular microcirculation seems promising as a potential biomarker for systemic microcirculation health.

The eye comprises two distinct vascular compartments with unique flow regulation mechanisms, making the evaluation of ocular microcirculation particularly intriguing (29). OCTA enables the convenient measurement of various parameters of the retina and choroid, providing real-time monitoring of blood flow changes. The retina typically maintains a consistent blood flow, independent of alterations in perfusion pressure, blood gas tension, or intraocular pressure. It is an internal autoregulatory function because the potential influence of autonomic innervation may be excluded (29, 30). By contrast, the choroidal circulation is regulated by extrinsic autonomic innervation. Choroidal blood flow reduction occurs due to efferent sympathetic nerve activation, leading to noradrenaline release and subsequent alpha 1-adrenergic receptor stimulation on vascular smooth muscle cells (29). The assessment of choroidal parameters appears highly dependable, given the assumed poor autoregulation of choroidal blood flow and the significant impact of ocular blood flow on choroidal characteristics. The choroid may be particularly susceptible to changes occurring during HD, and serves as an indicator of microcirculatory status and its response to HD treatments (31, 32).

In our study, significant decreases were observed in the retinal vasculature (SVD, VD) surrounding the macular zone and optic nerve, aligning with the findings of Coppolino et al. (31) who specifically noted a significant reduction in the deep capillary plexus (DCP: Whole, fovea, and parafovea) toward the end compared to the beginning of HD. Conversely, Zegrari et al. (14) reported no notable changes in vascular density within the retinal vasculature. Maharshak et al. (33) claimed that the retinal nerve fiber layer decreased after HD, but the difference was not statistically significant. The reduction in choroidal thickness, vascular density, and perfusion area may be correlated with weight loss, decreased serum osmolarity, and reduced systolic blood pressure (BP). Possible factors contributing to these differing results include variations in sample sizes, age, gender, underlying diseases, as well as differences in measurement intervals and types of OCTA machines used.

The parameters we assessed, including PA, retinal inner VD, and CVI, exhibited significant decreases after HD in multiple quadrants, particularly notable in the inferior quadrant. During ultra-wide-angle SS-OCTA scanning, the upper and lower areas are susceptible to the patient’s eyelids and eyelashes, which may cause partial coverage artifacts that affect the perfusion density. It is hypothesized that the diagnostic potential of the inferior quadrant may surpass that of all other OCTA measurements in the HD group, mirroring observations in glaucoma eyes (34). Nevertheless, potential confounding factors such as patient non-compliance, motion artifacts, and positional variations cannot be disregarded. Notably, the decline in CVI in the optic disk area post-HD was more pronounced compared to the macular region. This divergence suggests that alterations in blood flow within the optic disk and choroidal layers are more susceptible, potentially serving as early indicators of systemic hemodynamic changes. Notably, the broader scanning range offered by SS-OCTA allows for thorough exploration of the peripheral retina and choroid (35, 36). This advantage might help us understand the progression of systemic diseases in advance. Shoshtari et al. (11) found that changes in the thickness of the 1,000-micron temporal and nasal choroidal regions before and after hemodialysis were significant. Furthermore, a meta-analysis by researchers (37) unveiled a notable decrease in subfoveal choroidal thickness (SCT) post hemodialysis, indicating a significant reduction among patients with ESRD. Wide-field SS-OCTA shows great promise in the assessment of microcirculation and comprehension of the pathophysiology of systemic vascular diseases.

There are a few limitations in the present study. Firstly, this is a cross-sectional study, fundus blood flow parameters were measured only before and after a single HD session. Long-term follow-up and the observation of dynamic changes are imperative for a more comprehensive understanding. Secondly, the identified indicators are susceptible to various factors, including motion artifacts, cooperation of patients and dioptric media opacity. To mitigate these confounding influences, we conducted multiple measurements and excluded patients with significant lens opacity. Furthermore, a larger sample size is required in future studies to obtain more reliable and robust results. It is worth noting that this study did not differentiate between different causes of ESRD. Antihypertensive medications, such as angiotensin-converting enzyme inhibitors, might have different effects on retinal vessels and blood flow.

## Conclusion

In conclusion, notable reductions in blood flow indicators were found after HD. The indicators of the choroid and optic disk area exhibited more pronounced decreases when compared to the inner retina and macular zone. Contrast administration did not be required to assessment of blood vessels by SS-OCTA, which is meaningful for patients with kidney failure. SS-OCTA emerges as a promising imaging technique for the timely detection of fundus alterations in hemodialysis patients.

## Data Availability

The raw data supporting the conclusions of this article will be made available by the authors, without undue reservation.
