# Supplementary material for: Retinal and choroidal blood flow changes in dialysis patients assessed by wide-field swept-source optical coherence tomography angiography
Source: Front Med (Lausanne). 2025 May 14;12:1524503. doi: 10.3389/fmed.2025.1524503 (PMC12116474; doi:10.3389/fmed.2025.1524503)
Supplement: Supplementary file 1 [file Data_Sheet_1.docx]

**Supplementary Table 1.**  Comparison of PA between groups in the macular region and optic region.

| PA | Macular area | | | Optic disc area | | |
| --- | --- | --- | --- | --- | --- | --- |
|  | Before HD | After HD | P | Before HD | After HD | P |
| Central ring | 0.785±0.003 | 0.781±0.022 | 0.340 | 0.031±0.087 | 0.018±0.028 | 0.084 |
| 3mm radius |  |  |  |  |  |  |
| Nasal | 1.572±0.004 | 1.562±0.048 | 0.270 | 1.323±0.167 | 1.312±0.133 | 0.185 |
| Temporal | 1.571±0.006 | 1.568±0.116 | 0.111 | 1.221±0.195 | 1.249±0.202 | 0.170 |
| Superior | 1.567±0.008 | 1.546±0.101 | 0.265 | 1.205±0.205 | 1.238±0.158 | 0.597 |
| Inferior | 1.571±0.003 | 1.551±0.096 | 0.005** | 1.273±0.159 | 1.243±0.163 | 0.039* |
| 6mm radius |  |  |  |  |  |  |
| Nasal | 5.301±0.009 | 5.271±0.096 | 0.024* | 5.239±0.261 | 5.261±0.104 | 0.753 |
| Temporal | 5.300±0.158 | 5.264±0.110 | 0.477 | 5.253±0.183 | 5.226±0.255 | 0.083 |
| Superior | 5.287±0.047 | 5.257±0.115 | 0.791 | 5.186±0.336 | 5.218±0.281 | 0.856 |
| Inferior | 5.300±0.004 | 5.282±0.061 | 0.002** | 5.273±0.11 | 5.259±0.173 | 0.071 |
| 9mm radius |  |  |  |  |  |  |
| Nasal | 7.969±0.546 | 7.945±0.482 | 0.698 | 8.613±0.790 | 8.688±0.368 | 0.724 |
| Temporal | 8.666±0.268 | 8.616±0.272 | 0.122 | 8.500±0.372 | 8.468±0.417 | 0.677 |
| Superior | 8.691±0.333 | 8.637±0.696 | 0.155 | 7.883±0.562 | 8.077±0.605 | 0.280 |
| Inferior | 8.605±0.393 | 8.527±0.424 | 0.016* | 8.808±0.088 | 8.723±0.502 | 0.048* |

**Supplementary Table 2.** Comparison of SVD between groups in the macular and optic region.

| SVD | Macular area | | | Optic disc area | | |
| --- | --- | --- | --- | --- | --- | --- |
|  | Before HD | After HD | P | Before HD | After HD | P |
| Central ring | 7.95±4.41 | 6.49±2.82 | 0.059 | 14.09±7.77 | 14.21±6.85 | 0.819 |
| 3mm radius |  |  |  |  |  |  |
| Nasal | 24.56±10.14 | 22.12±9.97 | 0.285 | 27.77±6.74 | 27.97±6.92 | 0.583 |
| Temporal | 22.33±8.7 | 20.96±8.37 | 0.158 | 29.37±7.77 | 31.52±4.88 | 0.174 |
| Superior | 25.19±10.54 | 22.4±10.74 | 0.054 | 19.04±6.28 | 19.32±6.14 | 0.255 |
| Inferior | 24.25±9.46 | 21.99±10.03 | 0.131 | 14.87±6.33 | 15.39±5.97 | 0.302 |
| 6mm radius |  |  |  |  |  |  |
| Nasal | 33.62±9.55 | 32.56±8.94 | 0.452 | 24.23±7.67 | 23.06±7.59 | 0.600 |
| Temporal | 15.89±5.61 | 14.97±5.81 | 0.128 | 37.41±8.17 | 34.74±8.35 | 0.193 |
| Superior | 24.96±7.71 | 22.16±7.31 | 0.059 | 31.77±6.33 | 31.06±6.82 | 0.594 |
| Inferior | 23.42±7.93 | 21.15±8.28 | 0.158 | 30.91±6.85 | 31.25±5.93 | 0.620 |
| 9mm radius |  |  |  |  |  |  |
| Nasal | 28.31±5.28 | 29.87±5.01 | 0.245 | 12.27±5.22 | 12.61±5.12 | 0.664 |
| Temporal | 10.3±2.74 | 9.84±3.23 | 0.260 | 25.38±9.91 | 22.84±10.08 | 0.035* |
| Superior | 19.72±5.65 | 19.29±6.93 | 0.666 | 17.50±4.49 | 18.88±6.91 | 0.371 |
| Inferior | 21.87±5.42 | 21.18±4.98 | 0.675 | 22.05±5.43 | 20.61±5.67 | 0.047* |

**Supplementary Table 3. Comparison of the retinal thickness and choroidal thickness before and after HD of Macular region**

| Macular | Retinal thickness | | | Choroidal thickness | | |
| --- | --- | --- | --- | --- | --- | --- |
|  | Before HD | After HD | *P* | Before HD | After HD | *P* |
| Central | 240.33±43.42 | 240.48±42.36 | 0.885 | 282.31±66.22 | 276.93±89.10 | 0.372 |
| 3mm radius |  |  |  |  |  |  |
| Nasal | 314.62±37.72 | 315.79±38.22 | 0.063 | 273.11±80.69 | 266.79±89.12 | 0.186 |
| Temporal | 309.62±41.21 | 309.48±40.69 | 0.859 | 297.21±73.88 | 293.64±82.98 | 0.177 |
| Superior | 316.74±37.38 | 314.4±37.97 | 0.317 | 295.12±73.91 | 283.07±87.59 | 0.036* |
| Inferior | 315.45±37.16 | 316.07±36.09 | 0.471 | 287.02±83.19 | 275.36±87.84 | 0.021* |
| 6mm radius |  |  |  |  |  |  |
| Nasal | 293.81±30.81 | 292.05±34.44 | 0.490 | 231.12±74.99 | 223.24±88.36 | 0.106 |
| Temporal | 272.5±44.24 | 270.79±42.33 | 0.087 | 285.38±74.74 | 277.69±76.53 | 0.016* |
| Superior | 282.36±27.95 | 281.17±27.76 | 0.316 | 287.64±71.70 | 275.36±74.03 | 0.001* |
| Inferior | 265.24±27.34 | 265.1±26.31 | 0.803 | 264.05±85.35 | 254.05±83.33 | 0.005* |
| 9mm radius |  |  |  |  |  |  |
| Nasal | 310.64±34.56 | 307.74±32.93 | 0.238 | 195.21±58.57 | 188.19±64.93 | 0.015* |
| Temporal | 229.02±30.29 | 228.31±30.00 | 0.45 | 261.05±61.33 | 253.81±58.90 | 0.046* |
| Superior | 255.38±17.28 | 255.33±20.07 | 0.98 | 283.17±65.70 | 275.69±63.45 | 0.002* |
| Inferior | 241.51±20.86 | 242.07±19.65 | 0.704 | 241.67±67.23 | 236.85±63.50 | 0.003* |

**Supplementary Table 4. Comparison of the central choroidal thickness before and after HD**

| Optic disc | Retinal thickness | | | Choroidal thickness | | |
| --- | --- | --- | --- | --- | --- | --- |
|  | Before HD | After HD | *P* | Before HD | After HD | *P* |
| 3mm radius |  |  |  |  |  |  |
| Nasal | 362.55±45.19 | 363.69±47.16 | 0.691 | 136.52±39.51 | 130.93±35.00 | 0.141 |
| Temporal | 314.79±48.47 | 316.29±52.15 | 0.333 | 130.98±46.98 | 124.29±38.84 | 0.179 |
| Superior | 405.17±51.46 | 408.45±49.23 | **0.011*** | 137.48±39.67 | 136.4±37.89 | 0.815 |
| Inferior | 391.24±48.31 | 394.71±46.87 | 0.129 | 127.41±38.24 | 125.71±43.08 | 0.297 |
| 6mm radius |  |  |  |  |  |  |
| Nasal | 293.81±30.81 | 292.05±34.44 | 0.49 | 190.93±57.53 | 182.10±54.01 | 0.070 |
| Temporal | 272.5±44.24 | 270.79±42.33 | 0.087 | 207.14±74.61 | 197.33±70.40 | 0.091 |
| Superior | 282.36±27.95 | 281.17±27.76 | 0.316 | 204.48±53.31 | 195.86±51.74 | **0.006*** |
| Inferior | 265.24±27.34 | 265.1±26.31 | 0.803 | 183.17±51.78 | 175.50±52.17 | **0.013*** |
| 9mm radius |  |  |  |  |  |  |
| Nasal | 310.64±34.56 | 307.74±32.93 | 0.238 | 211.45±53.61 | 202.36±55.39 | **<0.001*** |
| Temporal | 229.02±30.29 | 228.31±30.04 | 0.45 | 246.50±65.12 | 239.88±67.76 | **0.015*** |
| Superior | 255.38±17.28 | 255.33±20.01 | 0.98 | 220.55±40.12 | 214.74±44.75 | **0.004*** |
| Inferior | 241.50±20.86 | 240.79±22.21 | 0.358 | 192.38±48.13 | 181.36±46.75 | **0.002*** |
